# Echocardiographic photorealistic imaging of leiomyomatosis intravascularis

**DOI:** 10.1007/s10554-020-01951-0

**Published:** 2020-08-02

**Authors:** Paweł Czub, Monika Budnik, Franciszek Majstrak, Janusz Kochanowski, Piotr Hendzel, Grzegorz Opolski

**Affiliations:** Medical University of Warsaw, Warsaw, Poland

**Keywords:** 3D echocardiography, Photorealistic imaging, Right atrial tumour

## Abstract

We present a case of patient with leiomyomatosis intravascularis. To diagnose, we used 3D photorealistic imaging which allowed us better visualisation of pathological structures as well as planning the surgical procedure in greater detail. The main advantage of this technique is the possibility to move the virtual light source and thus visualize the specific part of the heart.

**Electronic supplementary material:**

The online version of this article (10.1007/s10554-020-01951-0) contains supplementary material, which is available to authorized users.

A 39 year old female patient was admitted to the hospital on suspicion of additional mass in the right atrium (RA). 2 weeks before, she underwent surgery to remove the tumours of the uterus and the bladder.

Transthoracic echocardiography confirmed the presence of inhomogeneous and mobile mass in the RA (Fig. 1a). Moreover, the inferior vena cava (IVC) was also involved. We decided to perform transesophageal echocardiography (TEE) which showed that the structure protruds across the tricuspid valve throughout the cardiac cycle (Fig. 1b). Using the latest display method, the Philips cardiac TrueVue 3D photorealistic imaging, we could see in detail a huge mass that was elongated, mobile, inhomogeneous, with no adhesion to the wall of the heart and the veins and vibrating along with the heartbeat (Fig. 1c, d, Supplemental Videos). The patient underwent a surgery, whereby a huge mass of about 30 cm in length was excised through RA on beating heart with cardiopulmonary bypass (Fig. 1e). Following the surgery, a histopathological examination diagnosed leiomyomatosis.

**Fig. 1:**
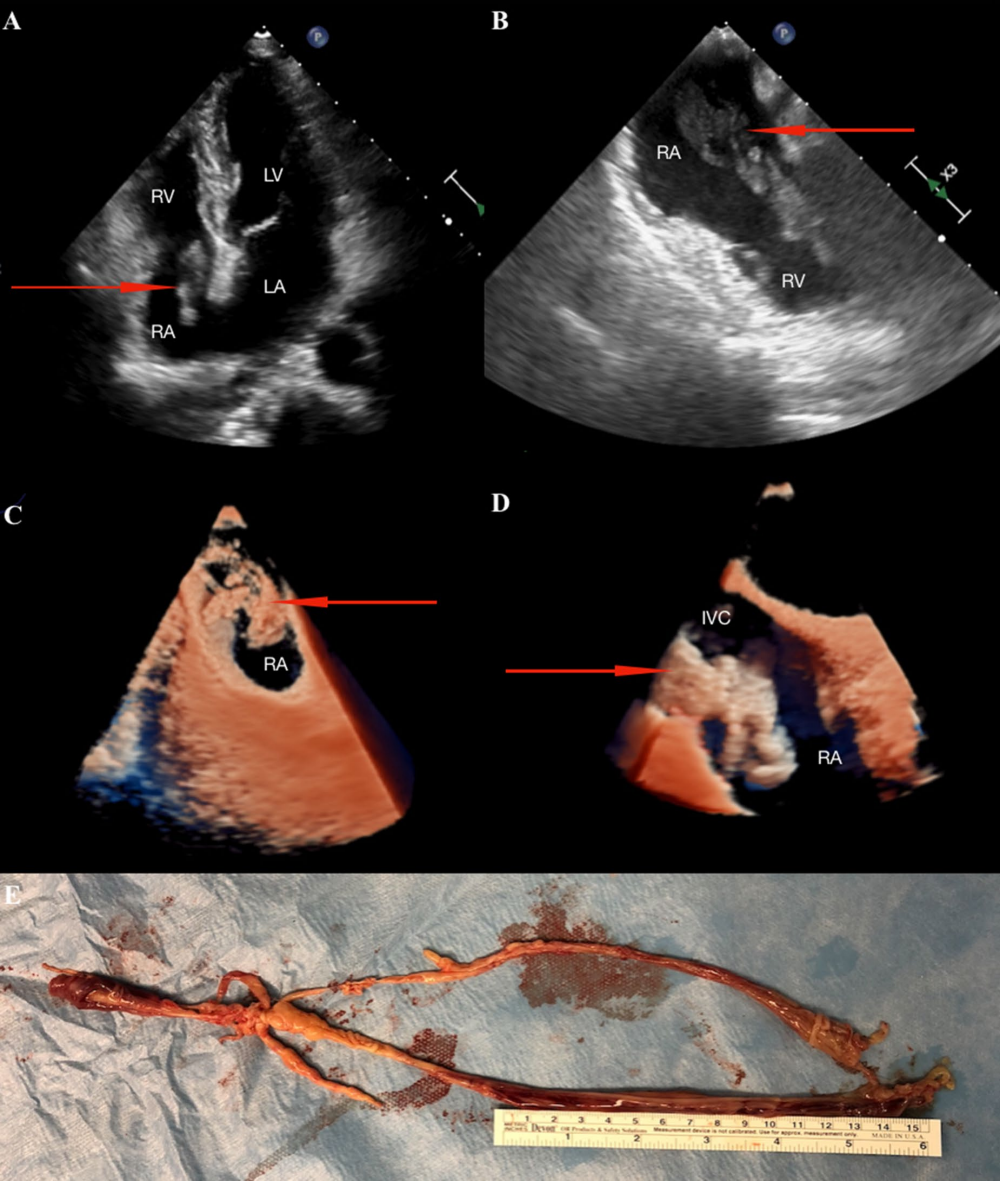
**a** TTE; 2D four chamber view; **b** TEE; 2D two chamber view; **c**, **d** TEE, 3D photorealistic imaging; **e** removed structure from RA and IVC. Arrows show the mass in the RA and IVC. *IVC* inferior vena cava, *LA* left atrium, *LV* left ventricle, *RA* right atrium, *RV* right ventricle

The use of 3D photorealistic imaging enables not only to better visually identify pathological structures, but also plan the surgical procedure in greater detail. Its main advantage over 3D echo is that it enables to move the virtual light source and thus visualize the specific part of the heart, especially the prosthetic valves and additional masses in the heart.

## Electronic supplementary material

Below is the link to the electronic supplementary material.

Supplementary material 1 (MOV 10062 kb)

Supplementary material 2 (MOV 8190 kb)

